# Mobile-First Web Access and Captioned Video in Francophone Cardiology Education: Multicountry Ecological Learning Analytics Study

**DOI:** 10.2196/90345

**Published:** 2026-09-08

**Authors:** Thomas Roussel, Elodie Benhaim, Louis Perrard, Benoit Merat, Shamir Vally, Remi Girerd, Pierre Deharo, Louis-Marie Desroche, École Numérique de Cardiologie, Louis-Marie Desroche

**Affiliations:** 1 Cardiology Department, Assistance Publique Hôpitaux de Marseille, Marseille, Provence-Alpes-Côte d'Azur, France; 2 Department of Research and Innovation, University Hospital of La Réunion, Saint Paul, Réunion, Réunion; 3 Ecole Numérique de Cardiologie (ENC), Saint Denis, La Réunion, France; 4 Cardiology Department, Centre Hospitalier Universitaire Amiens-Picardie, Amiens, Hauts-de-France, France; 5 Cardiology Department, Hôpital d'instruction des Armées Percy, Clamart, Île-de-France, France; 6 Cardiology Department, University Hospital of La Réunion, Saint Denis, La Réunion, France; 7 Emergency Department, University Hospital of La Réunion, Saint Pierre, La Réunion, France; 8 Réunisim, University Hospital of La Réunion, Saint Pierre, La Réunion, France; 9 Cardiovascular and Nutrition Research Center (C2VN), INSERM U1263, INRAE U1260, Aix-Marseille Université, Marseille, Provence-Alpes-Côte d'Azur, France; 10 Cardiology Department, University Hospital of La Réunion, Allée des topazes CS 11 021, Saint Denis, La Réunion, 97400, France, 33 692889708; 11 Clinical Investigation Center – Epidemiology and Clinical Research (CIC-EC), INSERM 1410, University Hospital of La Réunion, Saint Denis, La Réunion, France; 12 See Acknowledgments

**Keywords:** mobile health, cardiology, medical education, health equity, digital divide, YouTube, learning analytics, ecological study

## Abstract

**Background:**

Mobile health (mHealth) and online video are increasingly central to cardiology education and point-of-care decision support. However, little is known about how simple design choices, such as mobile-first web layouts and captioned videos, translate into real-world practice across countries with different income levels.

**Objective:**

This exploratory ecological study used routinely collected, cross-platform learning analytics from a francophone cardiology mHealth initiative to (1) describe how mobile web access and caption-enabled YouTube viewing varied across World Bank income groups and (2) examine whether greater reliance on mobile access was associated with poorer engagement on the website or on YouTube.

**Methods:**

We analyzed country-level analytics from the École Numérique de Cardiologie (ENC; Saint-Denis) mobile-optimized website and its companion YouTube channel (YouTube, LLC [Google LLC]) over a two-year window (September 2023 to September 2025). Countries were grouped as high-, middle-, or low-income (World Bank, three-level classification). Country-level metrics included mobile device session share; website bounce rate; time on page; and YouTube average view duration, audience retention, and intentional views. Caption-related and demographic YouTube metrics were available only as income-group aggregates and were therefore reported descriptively as between-group contrasts; country-level inferential analyses were restricted to country-level variables. Reporting followed the STROBE (Strengthening the Reporting of Observational Studies in Epidemiology) checklist.

**Results:**

Thirty-four countries contributed data: 13/34 (38%) high-income, 14/34 (41%) middle-income, and 7/34 (21%) low-income. Caption-enabled watch time was 18.8% in high-income countries (HICs), compared with 38.7% in middle-income countries (MICs) and 60.9% in low-income countries (LICs), representing a caption equity gap (CEG) of 42.1% between low- and high-income settings. Median website mobile share rose with decreasing income (36.5%, 63.3%, and 81.4%, respectively; Jonckheere-Terpstra *P=*.01). Across income groups, higher caption-enabled watch time coincided with a higher share of intentional views. At the country level, greater reliance on mobile access was not associated with higher bounce rate or shorter time on page, and Spearman correlations between mobile share and YouTube engagement metrics were small and nonsignificant (all |ρ|≤0.28; all *P*≥.18).

**Conclusions:**

In this multicountry, francophone, mHealth learning analytics case study, mobile web access and captioned video were used most intensively in lower-income settings, and greater reliance on mobile access was not associated with measurable penalties in basic engagement metrics. These findings support treating mobile-optimized design and systematic captioning as core, low-cost, access-supporting features for equitable digital cardiology education. They also suggest that routinely collected platform indicators can serve as practical equity-monitoring signals for global mHealth initiatives, while underscoring that engagement metrics are not direct measures of learning or behavior change.

## Introduction

Digital technologies are reshaping cardiovascular education, and many societies and academic centers now deliver teaching through online and hybrid formats alongside mobile health (mHealth) initiatives [1-4]. Within this ecosystem, online video, particularly YouTube (LLC [Google LLC]), has become a major channel for professional and patient education, although its educational contribution remains only partly characterized and is rarely examined using real-world usage data [5]. Experimental studies suggest that short videos and digital cardiology resources can improve knowledge, retention, and patient-reported outcomes [6-8].

These opportunities intersect with persistent inequities. The burden of cardiovascular disease is greatest in low-income countries (LICs) and middle-income countries (MICs), where access to guideline-based care, connectivity, and continuing education remains limited [9-12]. Global connectivity reports describe a large “usage gap”: despite widespread mobile broadband coverage, many clinicians work with unstable electricity, constrained bandwidth, and a strong dependence on personal smartphones as their primary digital device for work and learning [11-13]. In such settings, mobile-first web design and lightweight content are not optional refinements but practical access requirements.

Subtitles and transcripts represent another simple access enabler for video. Same-language captions benefit many learners, including second-language users and people who are deaf or hard of hearing, and automatically generated subtitles can support learning when sufficiently accurate [14,15]. On public platforms such as YouTube, captions and automatic translation could extend the reach of single-language cardiology content across income settings. However, little is known about how often subtitles are actually used in medical or cardiology education, how this use varies with country income, or how it relates to mobile access.

We therefore conducted an exploratory, multicountry ecological analysis of routinely collected learning analytics from a francophone, mobile-first cardiology education website and its YouTube channel. Our two prespecified objectives were (1) to describe, across World Bank income groups, the share of website sessions from mobile devices and the share of YouTube watch time with subtitles enabled and, (2) using only variables measured at the country level, to examine whether greater reliance on mobile access was associated with poorer engagement on the website or on YouTube. We hypothesized that both access indicators would be higher in MICs and LICs than in high-income countries (HICs) and that greater reliance on these access enablers would not be associated with poorer engagement metrics.

## Methods

### Study Design and Setting

This study was an exploratory, multicountry ecological analysis of routinely collected analytics from a French-language cardiology education website [16] and its companion YouTube channel. The website is an open-access cardiology education resource providing clinical protocols, instructional videos, and practical tools covering diverse cardiology topics. The country was selected as the natural unit of analysis because (i) the World Bank income classification used as the main stratification variable is defined at the country level, and (ii) École Numérique de Cardiologie (ENC; Saint-Denis) platform analytics aggregate user activity at the country level. The ecological design is appropriate for an equity-focused descriptive question; we did not seek to make individual-level inference and acknowledge in the Discussion that ecological associations cannot establish individual-level effects.

All metrics were aggregated over a prespecified two-year window (September 2023 to September 2025). The website was designed using a mobile-first responsive layout: core pages and PDFs were optimized and tested on smartphones (large tap targets, simplified navigation, lightweight media) to support use on small screens and variable bandwidth. No individual-level data were accessed.

### Data Sources

#### Website Analytics

Website analytics were exported from Fathom Analytics (Conva Ventures Inc.), a privacy-focused web analytics platform. For each country, we obtained the number of sessions, unique visitors, and page views, traffic sources (organic search, direct, social, other), bounce rate, and average time on page. Device information was used to calculate the percentage of sessions from mobile devices among sessions with a known device type and the percentage of sessions with an unknown device type. We define mobile devices as smartphones and tablets combined, and this definition is used consistently in Methods, Tables, and Figure captions. For the main content categories (clinical protocols, practical tools, video pages), we also computed the percentage of sessions from mobile devices.

#### YouTube Analytics

For each country, we extracted the number of views, total watch time, average view duration, audience retention, the percentage of viewers continuing beyond an introductory segment, views per viewer, and the number and percentage of views classified by the platform as intentional. Intentional views are defined by YouTube as views that the platform classifies as user-driven (eg, search-, suggestion-, or subscription-driven views) rather than impressions or auto-played starts. We selected this metric as a complement to total views because it captures viewer intent and is therefore a meaningful indicator of voluntary engagement with educational content.

A key measurement caveat applies to the caption and demographic indicators introduced here: unlike the country-level website and YouTube metrics described above, they were not available at the country level. Caption-related YouTube metrics (percentage of watch time with any subtitles enabled and percentage with French subtitles) and summary viewer demographics (dominant age band and percentage of male viewers) were available in YouTube Studio only at aggregated geographic levels that corresponded approximately to World Bank income groups, not to individual countries. For each income group (high-, middle-, and low-income) we exported these caption and demographic indicators and assigned the corresponding group-level values to all ENC countries in that group, so that web and YouTube indicators could be tabulated together. As a direct consequence, these caption-related and demographic variables took only three distinct values across the 34-country dataset and showed no within-group variability.

### Country Income Classification

Countries were mapped to the World Bank’s 2024‐2025 four-level income classification (high-, upper-middle, lower-middle-, and low-income economies), a widely used framework that enables standardized cross-country comparisons and reflects major disparities in digital infrastructure, resource availability, and access conditions that may influence engagement with digital health education interventions. For main analyses, upper- and lower-middle-income categories were collapsed into a single middle-income group, yielding a three-level ordinal variable (high, middle, and low income); the four-level classification was retained for descriptive sensitivity analyses.

### Outcome and Access Definitions

Primary access enabler indicators were website mobile access (percentage of sessions from mobile devices among sessions with a known device type) and YouTube caption use (percentage of watch time with any subtitles enabled and any language). Country-level secondary website outcomes, used as country-level proxies of engagement, were bounce rate and average time on page. Country-level secondary YouTube outcomes were average view duration, audience retention, viewer retention beyond the introductory segment, views per viewer, and percentage of intentional views. We also summarized, by income group, mobile share within each website content category, referrer structure, LinkedIn (LinkedIn Corporation) followers and visitors, and website unique visitors; these latter metrics were considered exploratory.

Caption-related YouTube metrics and demographic YouTube metrics were available only at the income-group level. We therefore treated them, throughout the analysis, as descriptive between-group contrasts and we did not include them in country-level inferential analyses. Bounce rate, time on page, average view duration, retention, viewer retention beyond the introductory segment, and intentional views are platform-defined engagement metrics; we explicitly note in the discussion that they are not direct measures of learning, comprehension, implementation, or behavior change. Demographic indicators (dominant viewer age band and percentage of male viewers) were derived from small YouTube Studio aggregates of logged-in viewers with available age and sex data and are descriptive only. In particular, the 100% male value in the low-income group reflects a very small aggregate and does not warrant inferential interpretation.

### Data Preprocessing

All analyses were conducted at the country level. Raw exports from each platform were imported into a unified database, nonnumeric placeholders were removed, and percentage variables were placed on a 0‐100 scale where needed. For YouTube, country-level indicators (views, watch time, average view duration, retention, viewer retention beyond the introductory segment, views per viewer, and intentional views) were retained as such. Caption-related indicators (percentage of watch time with any subtitles enabled and percentage with French subtitles) and viewer demographics (age band code, percentage of male viewers) were available only at the income-group level; the same group-level values were therefore shared by all countries within a given income group, and there was no within-group variability in these indicators. For YouTube data, we retained all countries with at least one record and a nonmissing income group. When multiple rows were present for a given country and platform, we computed country-level medians over the observation window. For cross-domain analyses, website and YouTube tables were merged on country and income group so that each country contributed a single combined record. Country-level YouTube engagement metrics (average view duration, audience retention, intentional views, and total views) were included for a given country only when YouTube Studio exported a nonmissing country-level value for that metric over the observation window. Low-volume country accounts without an available country-level value were excluded pairwise. Country-level correlations involving these metrics were therefore based on 24 countries (9 high-, 9 middle-, and 6 low-income; 23 countries for audience retention, with one fewer low-income country), compared with all 34 countries for website-only correlations.

### Statistical Analysis

Given the modest sample size and skewed country-level distributions, analyses were prespecified as exploratory and relied on nonparametric tests and robust regression, with emphasis on effect sizes and ordered gradients. For variables measured at the country level (website mobile share, bounce rate, time on page, and country-level YouTube engagement indicators), we examined gradients across income groups using the Kruskal-Wallis test and tested for a monotonic trend with the Jonckheere-Terpstra test under the prespecified order high<middle<low income. Medians and IQRs by income group are reported for all main metrics. Because most reported percentages are country-level metrics (eg, percentage of sessions from mobile devices and percentage of watch time with captions) that are then summarized as a median or aggregate across countries, rather than proportions computed from a single numerator and denominator, a single absolute count cannot be attached to each percentage; where a discrete count and total are available (eg, number of countries per income group, sample sizes for correlations and regressions, numbers of visitors, views, or followers), both the count and the percentage or total are reported. Country-level Spearman rank correlations were computed between mobile share and engagement outcomes (website bounce rate, time on page; country-level YouTube average view duration, retention, and intentional views). Because our hypotheses were directional (a monotonic gradient across ordered income groups), the Jonckheere-Terpstra trend test was prespecified as the primary test for these ordered comparisons; it is more sensitive to monotonic trends than the omnibus Kruskal-Wallis test, which does not assume an ordering. Where the two tests diverge, the trend test therefore takes precedence.

**Figure 1.**
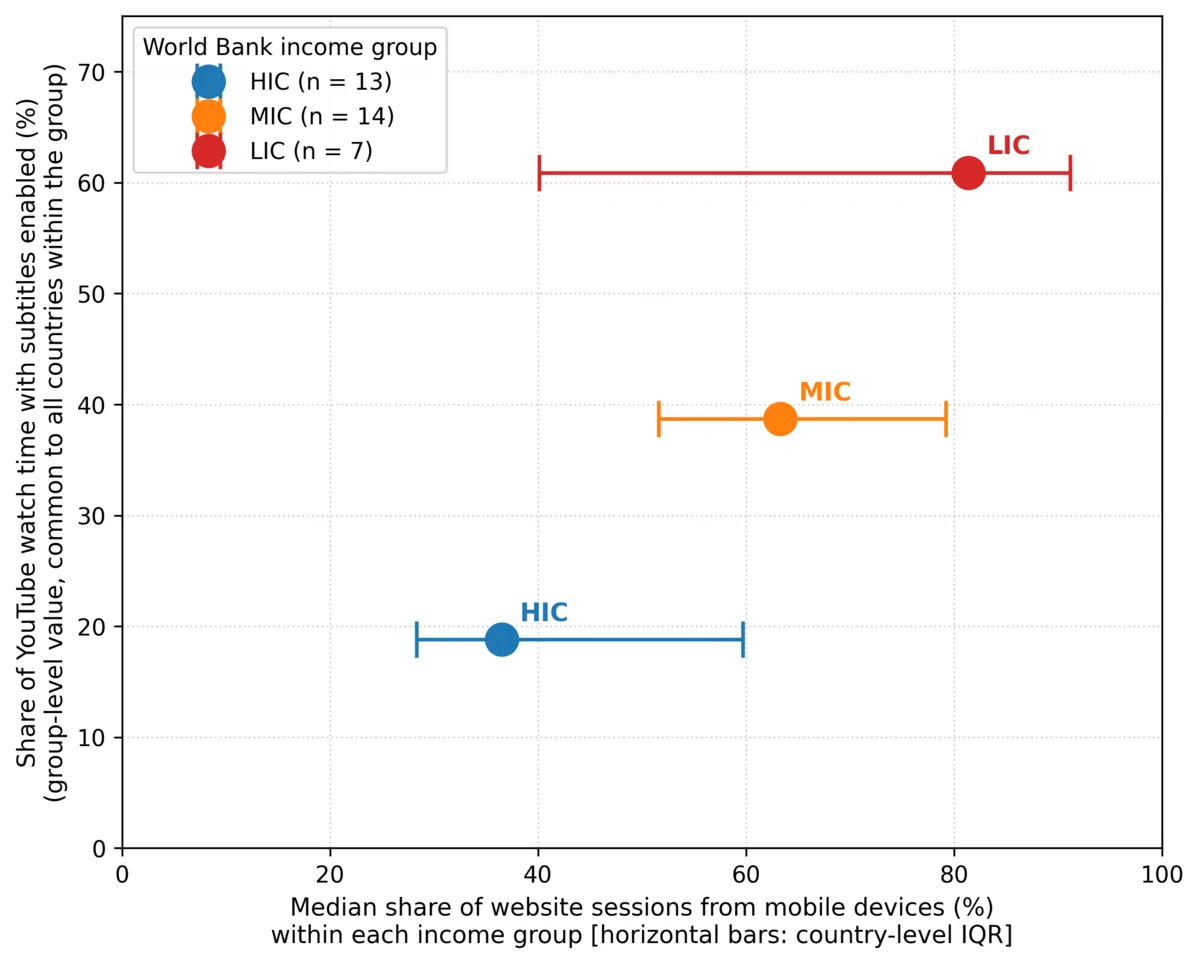
Cross-domain coherence between website mobile access and YouTube caption use, by income group (three points). Group-level summary of two access indicators across the three World Bank income groups: median share of website sessions from mobile devices (x-axis, country-level medians within each group, with horizontal bars showing the country-level IQR) and share of YouTube watch time with subtitles enabled (y-axis, group-level value common to all countries within the group). Points correspond to HIC (36.5%, 18.8%), MIC (63.3%, 38.7%), and LIC (81.4%, 60.9%). The figure shows that both access indicators increased monotonically from high- to low-income groups; because YouTube caption-enabled watch time was available only as one value per income group, this representation deliberately avoids a country-level scatter that could misleadingly suggest within-group caption variability. A country-level scatterplot of mobile share against the assigned group-level caption value is provided in Figure S1 in Multimedia Appendix 1, with an explicit note that all country dots within an income group share the same caption value. HIC: high-income country; LIC: low-income country; MIC: middle-income country.

Caption-related and demographic YouTube metrics, available only at the income-group level, were summarized descriptively as three between-group values; we did not compute country-level correlations or *P* values for these variables, and we did not include them in inferential models. To assess cross-domain coherence between platforms, we summarized, for each income group, the median website mobile share (computed across countries) and the income-group aggregate caption-enabled watch time and compared them descriptively (Figure 1, three-point income-group representation).

For website engagement we fitted median (*τ*=0.5) quantile regression models with bounce rate or average time on page as the outcome and income group plus website mobile share (proportion from 0 to 1) as predictors; interaction terms between income group and mobile share were explored in secondary models. LinkedIn followers, LinkedIn visitors, and website unique visitors were summarized by income group and compared using Kruskal-Wallis tests. Robustness of the main gradients was evaluated by repeating key comparisons under the alternative four-level income classification, in leave-one-country-out analyses, and in a sensitivity analysis excluding France (the dominant high-income contributor). *P* values are reported as *P=*.XX or *P<*.001; statistical significance was set at *P=*.05 (two-sided). All analyses were performed in R (R Foundation for Statistical Computing), version 4.5.0.

### Reporting Framework

This study is reported in accordance with the STROBE (Strengthening the Reporting of Observational Studies in Epidemiology) checklist for cross-sectional observational studies. The completed STROBE checklist is provided as Checklist 1.

### Ethical Considerations

The study used only aggregated, country-level web analytics without any directly or indirectly identifiable personal data and did not involve any intervention or contact with patients or health professionals. Under French law on research involving human participants (“loi Jardé”) [17] and the French Public Health Code [18], such secondary analyses of fully anonymized, aggregated data do not require review by a Comité de Protection des Personnes or individual informed consent. The project complied with applicable French and European data-protection requirements, including the General Data Protection Regulation.

## Results

### Country Sample

**Figure 2.**
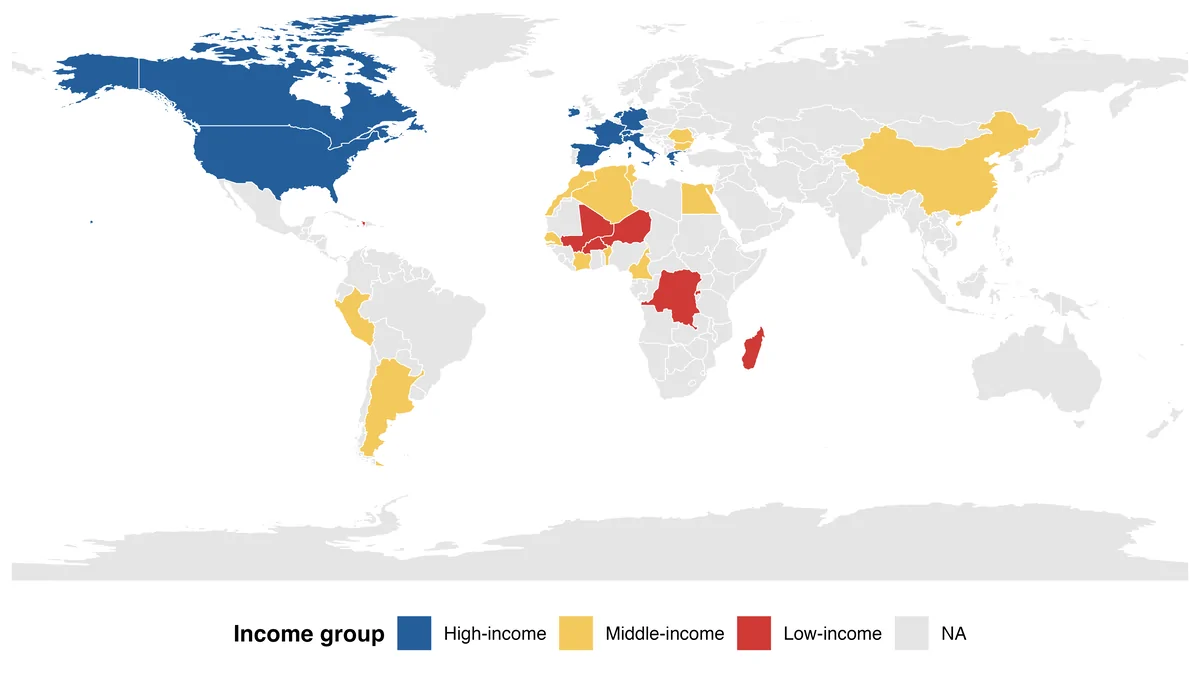
Included countries by World Bank income group. World map showing the 34 countries with École Numérique de Cardiologie (ENC) web and YouTube analytics, colored by World Bank income group (high-, middle-, or low-income). Countries without ENC data or without a mapped income category are shown in gray (not available [NA]). The map highlights a predominantly francophone yet geographically diverse reach spanning Europe, North America, Latin America, North Africa, Sub-Saharan Africa, and Asia over the fixed two-year observation window.

Web and YouTube analytics from the ENC were available for 34 countries: 13/34 (38%) HICs, 14/34 (41%) MICs, and 7/34 (21%) LICs. The audience was predominantly but not exclusively francophone, spanning Europe, North America, Latin America, North and Sub-Saharan Africa, and Asia (Figure 2).

### Income-Group Descriptive Contrasts: Caption Use and Viewer Demographics on YouTube

Caption-related and demographic YouTube metrics were available only as income-group aggregates and therefore took only three distinct values across the dataset; the contrasts below are presented as descriptive between-group differences without country-level *P* values or correlation coefficients.

Over the two-year study window, the ENC website attracted 34,340 unique visitors across 34 countries, generating 180,563 page views, while the companion YouTube channel accumulated 171,009 views and 7,451 hours of watch time.

**Figure 3.**
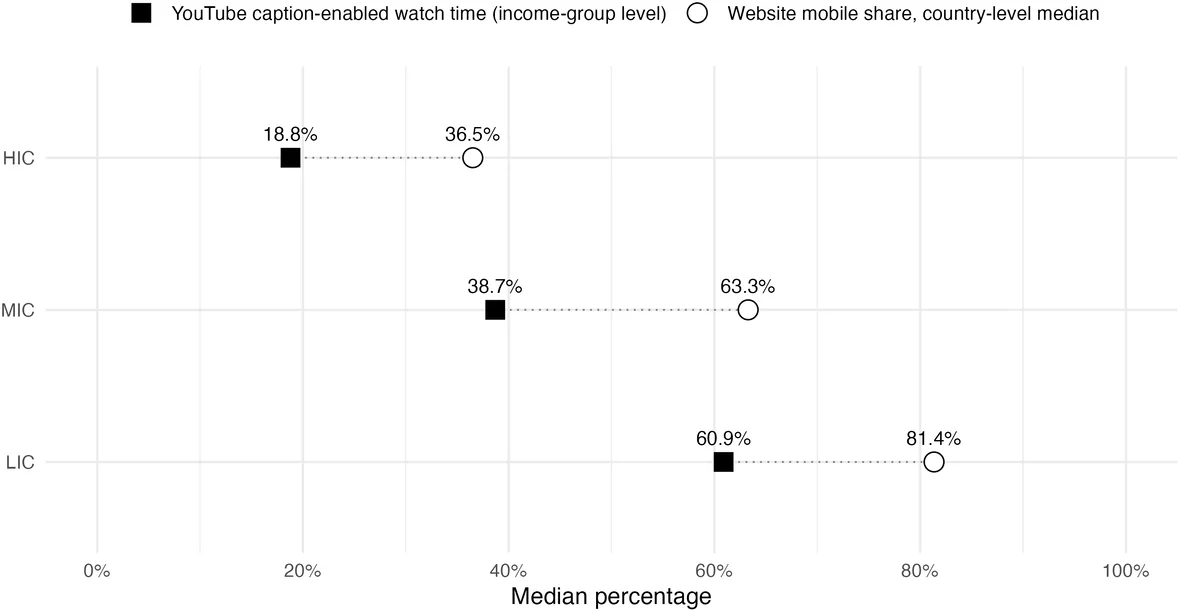
Access enablers by income group: YouTube captions and website mobile access. Income-group aggregate share of YouTube watch time with subtitles enabled (square marker, weighted by watch time within each income-group aggregate) and median share of ENC website sessions from mobile devices among sessions with a known device type (open circle) for each World Bank income group (HIC, MIC, LIC). The dotted line within each income group is provided for visual comparison only between two distinct indicators (caption-enabled watch time and website mobile share); it does not represent an uncertainty interval, a temporal change, or any modeled relationship. Caption-enabled watch time was 18.8%, 38.7%, and 60.9% in HICs, MICs, and LICs, respectively, corresponding to a caption equity gap of 42.1% between LICs and HICs. Website mobile share showed a similar gradient (36.5%, 63.3%, and 81.4%, respectively). Caption-related metrics were available only at the income-group level (one value per group, common to all countries in the group). HIC: high-income country; LIC: low-income country; MIC: middle-income country.

The income-group aggregate share of YouTube watch time with any subtitles enabled increased monotonically with decreasing income, from 18.8% in HICs to 38.7% in MICs and 60.9% in LICs (Table 1, Panel A; Figure 3). As a percentage of total watch time within each income-group aggregate, rather than an average across countries or videos, this share is inherently weighted by watch time. This corresponds to a caption equity gap (CEG) of 42.1% (60.9%-18.8%) between LICs and HICs. The share of watch time with French captions and with non-French captions varied across income groups (HICs: 2.3% French, 16.5% non-French; MICs: 0.3% French, 38.4% non-French; LICs: 17.5% French, 43.4% non-French), with non-French captions accounting for the largest share of caption-enabled watch time in middle- and low-income groups (Table 1, Panel A).

Across income groups, dominant viewer age band was older in HICs (≥65 years) than in MICs (25‐35 years) and LICs (45‐55 years); however, this pattern was not monotonic with caption-enabled watch time, as LICs had a higher caption-enabled watch time than MICs but an older dominant age band (Table 1, Panel A bis; see Limitations).

**Table 1.** Caption use, viewer demographics, mobile web access, and engagement by World Bank income group.^a^

Income group	Panel A^b^—income-group-level YouTube indicators			Panel A bis—income-group-level demographic indicators		Panel B^c^—country-level indicators, median (IQR); n countries					
	Caption-enabled watch time (any subtitles), %	French captions, %	Non-French captions, %	Dominant viewer age band, years	Male viewers, %	Mobile share, %	Bounce rate, %	Time on page, s	YouTube AVD^d^, min	YouTube retention, %	n countries
High-income (HIC^e^)	18.8	2.3	16.5	≥65	62	36.5 (28.3‐59.7)	65.5 (60.8‐68.5)	43.2 (39.2‐49.4)	2.30 (2.05‐2.82)	36.0 (27.0‐40.0)	13
Middle-income (MIC^f^)	38.7	0.3	38.4	25‐35	46	63.3 (51.6‐79.2)	58.5 (49.0‐66.3)	46.4 (33.8‐70.9)	2.55 (2.28‐2.78)	36.0 (34.0‐45.0)	14
Low-income (LIC^g^)	60.9	17.5	43.4	45‐55	100^h^	81.4 (40.1‐91.2)	51.2 (43.5‐59.0)	74.2 (52.8‐103.6)	3.56 (3.31‐4.44)	49.0 (37.0‐53.0)	7

a Demographic values are derived from small YouTube Studio aggregates of logged-in viewers and are descriptive only (see *Methods*, *Outcome and Access Definitions* section).

b Panel A reports YouTube indicators that were available only at the income-group level in YouTube Studio and were assigned to all countries within an income group; these values are therefore identical across all countries of the same group and are reported descriptively, without country-level *P* values or correlation coefficients.

c Panel B reports country-level medians and IQRs. Mobile share: percentage of website sessions from mobile devices (smartphones or tablets) among sessions with a known device type. Bounce rate: proportion of single-page sessions, expressed as percentage. Time on page: average time spent on a page (seconds). YouTube AVD: average view duration (minutes); retention: average percentage of each video watched. The “n countries” column applies to mobile share, bounce rate, and time on page (13 HICs, 14 MICs, 7 LICs; 34 total). YouTube AVD was available for a smaller subset of countries (9 HICs, 9 MICs, 6 LICs; 24 total) and YouTube retention for a further reduced subset (9 HICs, 9 MICs, 5 LICs; 23 total) (Table S1 in Multimedia Appendix 2 for per-metric denominators).

d AVD: average view duration.

e HIC: high-income country.

f MIC: middle-income country.

g LIC: low-income country.

h The low-income value is based on a very small aggregate; see *Method*s section.

### Country-Level Analyses: Mobile Web Access and Engagement

Website mobile share, measured at the country level, increased with decreasing income (Table 1, Panel B; Figure 3). The median mobile share of ENC website sessions was 36.5% (IQR 28.3‐59.7) in HICs, 63.3% (IQR 51.6‐79.2) in MICs, and 81.4% (IQR 40.1‐91.2) in LICs. The prespecified ordered trend test supported a monotonic increase in mobile share from HICs to MICs to LICs (Jonckheere-Terpstra *P*=.01; Kruskal-Wallis H=5.11; *P*=.08). The proportion of website sessions from devices of unknown type was negligible (median 0% in HICs, MICs, and LICs; range 0%‐0% in each group); no country exceeded the 40% threshold used for exclusion from mobile-share analyses, so the mobile-share denominator effectively comprised all sessions and could not materially bias the observed gradient.

### Country-Level Spearman Correlations Involving Mobile Share

Country-level Spearman correlations between website mobile share and country-level engagement outcomes were small in magnitude and not statistically significant (mobile share vs bounce rate: ρ=−0.05, *P=*.76, n=34; vs time on page: ρ=0.07, *P=*.71, n=34; vs YouTube average view duration: ρ=0.05, *P=*.80, n=24; vs YouTube audience retention: ρ=−0.03, *P=*.88, n=23; vs YouTube intentional views: ρ=0.28, *P=*.18, n=24). For reference, bounce rate and time on page were inversely correlated at the country level (ρ=−0.48, *P=*.004, n=34; Table 2).

**Table 2.** Country-level Spearman correlations between website mobile share and country-level engagement outcomes.^a^

Pair of country-level variables	Spearman ρ	*P* values	N
Website mobile share vs bounce rate	−0.05	.76	34
Website mobile share vs time on page	0.07	.71	34
Website mobile share vs YouTube average view duration (country-level)	0.05	.80	24
Website mobile share vs YouTube audience retention (country-level)	−0.03	.88	23
Website mobile share vs YouTube intentional views, % (country-level)	0.28	.18	24
Bounce rate vs time on page	−0.48	.004	34
Website mobile share vs YouTube total views (country-level)	−0.06	.79	24

a All variables are measured at the country level; correlations are based on country-level complete cases for each pair. Caption-related and demographic YouTube metrics are not included in this table because they were available only at the income-group level (see *Methods* and Table 1 Panel A); they are reported descriptively in the *Results*. Country-level YouTube metrics were available for 24 of 34 countries (high-income: 9/13; middle-income: 9/14; low-income: 6/7; audience retention: 23/34, with 5/7 low-income countries); website-only correlations include all 34 countries. Coverage was proportionally similar across income groups, making substantial selection bias unlikely.

### Median Quantile Regression for Web Engagement

In median (*τ*=0.5) quantile regression models for web engagement outcomes (Table 3), income group was associated with bounce rate but not with time on page. For bounce rate, the linear contrast for income (ordered from high- to low-income) was negative (β=−0.130; 95% CI −0.219 to −0.041; *P=*.01), consistent with lower median bounce rates in lower-income countries. The quadratic contrast was small and not statistically significant (β=−0.023; 95% CI −0.119 to 0.072; *P=*.64). For median time on page, neither the linear nor the quadratic income contrasts reached statistical significance (*β*=21.2 s, 95% CI –11.8 to 54.1, *P=*.22; *β*=12.6 s, 95% CI −18.6 to 43.8, *P=*.44). Website mobile share, modeled as a 0‐1 proportion, was not independently associated with either outcome (bounce rate: +0.072, 95% CI −0.128 to 0.272, *P=*.48; time on page:+16.0 s, 95% CI −44.8 to 76.8, *P=*.61). Overall, these analyses did not provide evidence that countries relying more heavily on mobile access experienced systematically worse website engagement.

**Table 3.** Median (*τ*=0.5) quantile regression of web engagement outcomes on income group (polynomial contrasts) and mobile share (n=34 countries).^a^

Outcome and predictor	Coefficient β	95% CI	*P* values
Median bounce rate (proportion of single-page sessions)			
Intercept	0.554	0.425 to 0.684	<.001
Income group (linear contrast)	−0.130	−0.219 to −0.041	.01
Income group (quadratic contrast)	−0.023	−0.119 to 0.072	.64
Website mobile share (proportion, 0‐1)	0.072	−0.128 to 0.272	.48
Median time on page (seconds)			
Intercept	47.1	9.7 to 84.5	.02
Income group (linear contrast)	21.2	−11.8 to 54.1	.22
Income group (quadratic contrast)	12.6	−18.6 to 43.8	.44
Website mobile share (proportion, 0‐1)	16.0	−44.8 to 76.8	.61

a Median (τ=0.5) quantile regression models were fitted at the country level for two web engagement outcomes: bounce rate (proportion of sessions with only one page viewed) and median time on page (seconds). Income group was entered as an ordered predictor using polynomial contrasts (high-, middle-, low-income); the “linear contrast” captures the overall gradient from high- to low-income, with negative coefficients indicating lower values in lower-income settings. Website mobile share was modeled as a proportion from 0 to 1 (0=0% of sessions on mobile; 1=100% on mobile), so the corresponding coefficients represent the expected change in the median outcome across this range. In these models, income group showed a modest association with bounce rate, whereas mobile share was not independently associated with either bounce rate or time on page (all *P≥.*48).

### Sensitivity Analyses

The income-related gradient in website mobile share was robust to several prespecified checks (Table S5 in Multimedia Appendix 3). In leave-one-country-out analyses, the Jonckheere-Terpstra trend test for mobile share remained significant in all 34 reestimations (all *P<*.05; largest *P=*.03). In a sensitivity analysis excluding France, the largest high-income contributor, the gradient was essentially unchanged (mobile share Jonckheere-Terpstra *P=*.02; Kruskal-Wallis H=4.94, *P=*.08). In median quantile regression models that added an income-by-mobile-share interaction, no interaction term reached statistical significance for either bounce rate or time on page (all *P>*.05; interaction coefficients and 95% CIs are reported in Table S5 in Multimedia Appendix 3)*,* providing no evidence of an interaction between income group and mobile share for either outcome; given the small sample size, this nonsignificant result does not establish that the absence of a mobile-access penalty is equivalent across income groups.

### YouTube Engagement Metrics by Income Group

Descriptively, country-level YouTube engagement metrics tended to be higher in lower-income groups (Table S1 in Multimedia Appendix 2). Median average view duration increased from 2.30 min in HICs to 2.55 min in MICs and 3.56 min in LICs, but neither the Kruskal-Wallis test (H=4.31, *P=*.12) nor the Jonckheere-Terpstra trend test (*P=*.06) reached statistical significance. Median audience retention rose from 36% (HICs) and 36% (MICs) to 49% (LICs), but Kruskal-Wallis and Jonckheere-Terpstra tests did not show statistically significant gradients (H=1.40, *P=*.50; Jonckheere-Terpstra *P=*.14).

### Cross-Domain Coherence Between Mobile Web Access and Caption Use

The income-group median of website mobile share and the income-group aggregate caption-enabled watch time both increased with decreasing income (Figure 1). To match the data structure, Figure 1 of the main paper shows one point per income group (HICs, MICs, LICs); a country-level scatterplot is provided Figure S1 in Multimedia Appendix 1 with an explicit note that caption-related values are common to all countries within an income group and that the country-level pattern therefore reflects between-group contrasts rather than within-group variability.

### Mobile Access by Content Category

Mobile reliance was high across all content categories outside high-income settings (Table 4, Table S3 in Multimedia Appendix 4). In HICs, medians for the share of mobile sessions were 37.7% for protocol pages, 40% for tool pages, and 52.2% for video pages, indicating relatively greater mobile use for video content. In MICs, medians increased to 66.1% for protocols, 60.5% for tools, and 77.1% for videos. In LIC mobile shares were 72.1% for protocols, 75% for tools, and 70% for videos. Even content typically consulted from a desktop in high-income settings (protocols, downloadable tools) was predominantly accessed from mobile devices in many MICs and LICs. The four-level World Bank classification yielded a broadly similar pattern, with mobile shares exceeding 60% in lower-middle- and low-income groups across all content categories and in upper-middle-income protocol and video pages, although upper-middle-income tool pages were lower (34.8%) (Table S3 in Multimedia Appendix 4).

**Table 4.** Mobile share of web sessions by content category and income group.^a^

Income group	Protocol pages — mobile sessions (% of known devices), median (IQR)	Tool pages — mobile sessions (% of known devices), median (IQR)	Video pages — mobile sessions (% of known devices), median (IQR)
High-income (HIC^b^)	37.7 (19.3-58.6)	40.0 (34.7-61.2)	52.2 (39.1-61.0)
Middle-income (MIC^c^)	66.1 (43.9-86.3)	60.5 (41.4-73.7)	77.1 (64.7-91.9)
Low-income (LIC^d^)	72.1 (44.3-95.6)	75.0 (31.2-88.5)	70.0 (56.6-90.6)

a For each country and content category (protocols, tools, videos), mobile share is the proportion of École Numérique de Cardiologie website sessions with a known device type that originated from mobile devices (smartphones or tablets). Values are medians across countries within each World Bank income group (three-level classification); only countries with at least one session in each category contribute to that median.

b HIC: high-income country.

c MIC: middle-income country.

d LIC: low-income country.

### Referrer Mix, LinkedIn Activity, and Website Reach

Referrer patterns varied by income group but were broadly dominated by organic and direct access (Table S2 in Multimedia Appendix 5 and Table S4 in Multimedia Appendix 6). At the country-median level, organic search accounted for 33% of sessions in HICs, 38.2% in MICs, and 43.5% in LICs; direct access accounted for 39.6%, 22.7%, and 20.5%, respectively. Social referrers contributed more in MICs (median 29.1%) than in HICs (14.3%) or LICs (12.3%). Median numbers of LinkedIn followers per country were 17 in HICs, 9 in MICs, and 5.5 in LICs, with no significant differences between income groups (H=3.19, *P=*.20). Median monthly LinkedIn visitors per country were also low across all income groups (H=0.01, *P=*.91). Median counts of unique ENC website visitors per country were highest in HICs (278), followed by MICs (93) and LICs (43; Table S2 in Multimedia Appendix 5). The country-level Spearman correlation between unique visitors and website mobile share was negative but not statistically significant (ρ=−0.28, *P=*.10, n=34).

## Discussion

### Principal Findings

In this exploratory ecological analysis of country-level learning analytics from a responsive francophone cardiology education website and its companion YouTube channel, we observed marked descriptive gradients in two practical access indicators across World Bank income groups. The share of website sessions from mobile devices and the share of YouTube watch time with captions enabled were substantially higher in middle- and especially LICs than in HICs, corresponding to a CEG of approximately 40% in caption-enabled watch time between low- and high-income settings. Because caption-related metrics were available only as income-group aggregates, these gradients should be interpreted descriptively rather than as precise country-level estimates, although the pattern was consistent and large. At the country level, greater reliance on mobile web access was not associated with measurable penalties in basic engagement metrics, including bounce rate, time on page, or country-level YouTube engagement indicators (all country-level Spearman |ρ|≤0.28; all *P≥*.18).

### Interpretation in Relation to Prior Work

These findings build on previous work on digital cardiology education, mHealth, and digital health equity. Major cardiology societies have emphasized the rapid growth of online congresses, courses, and blended training, and the central role of video in modular, on-demand learning [1-3,5-7]. Digital cardiology patient education interventions have likewise improved knowledge and patient-reported outcomes, supporting the value of carefully designed digital content beyond professional training alone [8]. Reviews and bibliometric analyses of digital and blended medical education describe the broader shift toward modular, on-demand formats integrated with video and social media [19-21]. At the same time, digital health frameworks and global connectivity reports highlight a persistent usage gap. Within this context, clinicians in LICs and MICs often rely on personal smartphones, unstable electricity, and constrained bandwidth [4,10-13]. The strong gradients we observed in mobile access and caption use are consistent with this picture of a digitally connected but resource-constrained workforce. Our study contributes to this literature in two ways. First, it links routine multicountry learning analytics to these equity concerns in a cardiology-specific, predominantly francophone context. Second, it focuses on modifiable design features, namely mobile-first website design and systematic captioning, rather than on bespoke mHealth applications alone.

Our results on subtitles align with experimental and applied work showing that captions can improve comprehension and information retention, particularly for second-language users and people who are deaf or hard of hearing [14,15]. Accessibility guidelines also identify captions, transcripts, and basic player controls as core requirements for inclusive media [22]. In our project, audio was in French and non-French subtitles relied substantially on YouTube automatic captioning and translation. Caption-enabled watch time is therefore likely to reflect a mix of creator decisions, platform features, and user activation. Despite these limitations, the higher caption-enabled watch-time share in lower-income groups suggests that subtitles may be an important access enabler for clinicians and trainees in these settings, even though we could not directly assess subtitle accuracy or individual learning outcomes. Taken together, our data suggest that mobile-optimized web access and captioned video may represent access-supporting features rather than trade-offs in basic engagement metrics.

### Implications for Design and Practice

From a design standpoint, these findings support treating mobile-optimized web layouts and captioned video as baseline features for cardiology education initiatives that seek international reach, rather than as optional enhancements. For clinicians and trainees in resource-constrained settings, responsive design, lightweight pages, and subtitles may reduce both technical and cognitive barriers to using educational material at the point of care [4,10,13,23]. Simple indicators such as mobile share, captioned watch time (including non-French captions), and geographic reach can be monitored as a low-cost equity dashboard alongside traditional metrics such as participation and satisfaction. For projects like ENC, practical next steps may include a lightweight mobile application with offline access to key protocols and tools, and a progressive professional translation of a core set of videos that builds upon, but does not rely solely on, automatic captioning. Professional societies and academic centers could similarly encourage contributors to provide mobile- and caption-ready content and to follow basic accessibility standards [22,24]. In HICs, the mobile gradient was milder, consistent with a dual-use pattern. Clinicians in these settings likely use desktop computers for prolonged work sessions involving protocols and tools, while relying on smartphones for quick lookups. In resource-constrained settings, however, clinicians and trainees appear to rely predominantly on smartphones as their primary device for both types of content.

### Engagement Metrics as Proxies for Educational Outcomes

Bounce rate, average time on page, average view duration, audience retention, viewer retention beyond the introductory segment, and intentional views are useful, routinely available platform metrics that provide insight into how frequently and for how long users engage with educational content. They are not, however, direct measures of learning, comprehension, implementation, or changes in clinical practice. On the one hand, longer time on page may reflect engagement, but it may also reflect slower mobile loading, divided attention, or difficulty understanding the content. On the other hand, a lower bounce rate may reflect interest, while it may also reflect a search for information that was not immediately found. Caption-enabled watch time indicates that subtitles were activated, but cannot determine whether the displayed text was accurate, comprehensible, or helpful to individual viewers. We therefore interpret these metrics as exploratory indicators of equitable reach and engagement rather than as proxies for educational outcomes. Linking these platform analytics with learner-level assessments of knowledge acquisition, perceived usefulness, and impact on clinical practice should be a priority for future research.

### Strengths and Limitations

This study has several strengths. It leverages routinely collected platform analytics from a real-world cardiology education initiative with a multicountry audience spanning high-, middle-, and low-income settings, and combines web and YouTube data from the same project to provide a rare cross-domain view of access enablers. Analyses were prespecified, used nonparametric methods appropriate for skewed country-level distributions, and focused on effect sizes and ordered gradients rather than solely on *P* values. By relying on simple indicators that are available on most web and video analytics dashboards, the approach is readily transferable to other mHealth and education projects with limited evaluation resources.

Important limitations must be acknowledged. The ecological design, with countries as the unit of analysis, precludes individual-level inference and is prone to ecological fallacy; we cannot identify which types of users drive the observed patterns or how usage relates to learning or clinical behavior. Caption-related and demographic YouTube metrics were available only as income-group aggregates and took only three distinct values across the dataset. They were therefore reported strictly as descriptive between-group contrasts, limiting the granularity of these analyses and precluding the inclusion of caption-related variables in country-level inferential models. The audience is self-selected and primarily francophone, which limits generalizability to other languages and likely underrepresents some large LICs and MICs. Our findings are therefore best interpreted as a francophone case study rather than a globally representative sample. Finally, web and platform metrics such as bounce rate, session duration, and caption-enabled watch time capture only limited aspects of engagement and are shaped by platform-specific definitions. In addition, the cross-sectional design did not allow us to examine temporal trends.

Country-level YouTube engagement metrics were available for 24 of 34 countries because YouTube Studio did not return stable country-level estimates for countries with fewer views. Coverage was proportionally similar across income groups (high-income 9/13, middle-income 9/14, low-income 6/7), making substantial selection bias unlikely. Nevertheless, the absolute number of LICs contributing YouTube metrics remained small. The association between caption use and viewer age was also not monotonic across income groups (older dominant age band in LICs than in MICs despite higher caption-enabled watch time in LICs), which tempers the equity narrative built around captions and should be interpreted cautiously.

### Conclusions

This exploratory ecological case study examined a responsive francophone cardiology education website and its companion YouTube channel. We observed marked descriptive gradients in mobile web access and caption-enabled video watch time across country income groups. The highest values were observed in lower-income settings. A CEG of approximately 40% was identified between LICs and HICs. At the country level, greater reliance on mobile devices was not associated with meaningful penalties in basic web engagement metrics. These findings are consistent with treating mobile-optimized design and systematic use of captions, including non-French subtitles, as core, low-cost, access-supporting components of equitable digital cardiology education, while emphasizing that platform engagement indicators are not direct measures of learning. Simple platform analytics may provide useful signals about global reach and inclusiveness and should, in future work, be linked with learner-level outcomes and evaluations of mHealth interventions.

## Supplementary material

10.2196/90345 Multimedia Appendix 1 Country-level scatterplot of website mobile share vs assigned income-group caption value. Country-level scatterplot of École Numérique de Cardiologie website mobile share (x-axis, percentage of sessions from mobile devices among sessions with a known device type) vs the income-group caption value assigned to that country (y-axis). Each point represents one country (n=34). Caption-enabled watch time was available only at the income-group level; therefore, all country points within a given income group share the same caption value (high-income country=18.8%, middle-income country=38.7%, low-income country=60.9%). The country-level pattern in this figure reflects between-group contrasts only, not within-group variability in caption use. We do not report country-level correlation coefficients on this figure.

10.2196/90345 Multimedia Appendix 2 YouTube engagement metrics (average view duration, audience retention, intentional views, total views) by World Bank income group, reported as country-level medians (IQR).

10.2196/90345 Multimedia Appendix 3 Prespecified, exploratory sensitivity analyses assessing the robustness of the income gradient in website mobile share, using percentile bootstrap (5000 resamples).

10.2196/90345 Multimedia Appendix 4 Mobile share of website sessions by content category (protocols, tools, videos) and four-level World Bank income group, reported as country-level medians.

10.2196/90345 Multimedia Appendix 5 Website referrer mix, LinkedIn activity, and website reach by World Bank income group (n=34 countries).

10.2196/90345 Multimedia Appendix 6 Website referrer mix by four-level World Bank income group, reported as country-level medians.

10.2196/90345 Checklist 1 STROBE checklist.

## Abbreviations

CEG: caption equity gap
ENC: École Numérique de Cardiologie
HIC: high-income country
LIC: low-income country
mHealth: mobile health
MIC: middle-income country
STROBE: Strengthening the Reporting of Observational Studies in Epidemiology
