# Supplementary material for: Mobile-First Web Access and Captioned Video in Francophone Cardiology Education: Multicountry Ecological Learning Analytics Study
Source: JMIR Mhealth Uhealth. 2026 Sep 8;14:e90345. doi: 10.2196/90345 (PMC13552991; doi:10.2196/90345)
Supplement: Supplementary file 1 [file mhealth-v14-e90345-s001.docx]

*Figure S1. Country-level scatterplot of ENC website mobile session share (x-axis) versus World Bank income-group caption-enabled watch time (y-axis; n=34 countries). Caption-enabled watch time was available only at the income-group level (HIC=18.8%; MIC=38.7%; LIC=60.9%); all countries within a group therefore share the same y-value, reflecting between-group differences only.*
