# Supplementary material for: Mobile-First Web Access and Captioned Video in Francophone Cardiology Education: Multicountry Ecological Learning Analytics Study
Source: JMIR Mhealth Uhealth. 2026 Sep 8;14:e90345. doi: 10.2196/90345 (PMC13552991; doi:10.2196/90345)
Supplement: Supplementary file 2 [file mhealth-v14-e90345-s002.docx]

**Table S1. YouTube engagement metrics by income group (country-level medians)**

| **Income group** | **Average view duration (minutes), median [IQR]** | **Audience retention (%), median [IQR]** | **Intentional views (%), median [IQR]** | **Total YouTube views per country, median [IQR]** | **Countries, n** |
| --- | --- | --- | --- | --- | --- |
| High-income (HIC) | 2.30 [2.05–2.82] | 36.0 [27.0–40.0] | 96.0 [93.2–100.0] | 90 [27–529] | 13 |
| Middle-income (MIC) | 2.55 [2.28–2.78] | 36.0 [34.0–45.0] | 100.0 [99.8–100.0] | 335 [46–2662] | 14 |
| Low-income (LIC) | 3.56 [3.31–4.44] | 49.0 [37.0–53.0] | 100.0 [100.0–100.0] | 40 [13–90] | 7 |

*Notes. Average view duration (AVD) is reported in minutes (corrected unit; see Methods). Audience retention is the average percentage of each video watched, as reported by YouTube analytics. Intentional views correspond to the YouTube-defined fraction of viewers reaching the intentional viewer threshold. Total YouTube views are summed views per country during the observation window. Values are country-level medians [IQR] across countries within each World Bank income group. The "Countries, n" column reflects the number of countries with at least one available YouTube metric in that group; per-metric n may differ slightly because of platform-side data availability for less-watched country accounts.*
