# Supplementary material for: Mobile-First Web Access and Captioned Video in Francophone Cardiology Education: Multicountry Ecological Learning Analytics Study
Source: JMIR Mhealth Uhealth. 2026 Sep 8;14:e90345. doi: 10.2196/90345 (PMC13552991; doi:10.2196/90345)
Supplement: Supplementary file 3 [file mhealth-v14-e90345-s003.docx]

**Table S5. Prespecified, exploratory sensitivity analyses for the income gradient in website mobile share**

| **Sensitivity analysis** | **Result** |
| --- | --- |
| Leave-one-country-out (34 re-estimations) | Jonckheere-Terpstra trend test for the mobile-share gradient remained significant in all 34 analyses (all *P<.05*; largest *P=.03*)*.* |
| Exclusion of France (largest high-income contributor; n=33) | Mobile share: Jonckheere-Terpstra *P=.02*; Kruskal-Wallis H=4.94, *P=.08*. Gradient unchanged. |
| Income × mobile-share interaction (median quantile regression) | No interaction term reached statistical significance for bounce rate or time on page (all *P>.05*). Bounce rate: income (linear contrast) × mobile share β=0.470, 95% CI −0.226 to 0.911, P=.28; income (quadratic contrast) × mobile share β=−0.245, 95% CI −0.954 to 0.601, P=.52. Time on page: income (linear contrast) × mobile share β=−37.1 s, 95% CI −156.6 to 122.6, P=.78; income (quadratic contrast) × mobile share β=−33.2 s, 95% CI −218.0 to 209.2, P=.95. |
| Four-level World Bank classification | Broadly similar pattern of gradients (see Multimedia Appendix Table S3). |

*Notes. Sensitivity analyses are exploratory and were computed with the same non-parametric pipeline as the primary analyses (R version 4.5.0). They assess the robustness of the prespecified mobile-share gradient; the main conclusions are unchanged. Interaction coefficients and 95% CIs were estimated via percentile bootstrap (5000 resamples), as asymptotic quantile-regression standard errors were unstable in this small, tied sample.*
