# Supplementary material for: Mobile-First Web Access and Captioned Video in Francophone Cardiology Education: Multicountry Ecological Learning Analytics Study
Source: JMIR Mhealth Uhealth. 2026 Sep 8;14:e90345. doi: 10.2196/90345 (PMC13552991; doi:10.2196/90345)
Supplement: Supplementary file 4 [file mhealth-v14-e90345-s004.docx]

**Table S3. Mobile share of web sessions by content category and four-level World Bank income group**

| **Income group (four-level)** | **Protocol pages — mobile sessions (% of known devices), median** | **Tool pages — mobile sessions (% of known devices), median** | **Video pages — mobile sessions (% of known devices), median** |
| --- | --- | --- | --- |
| High-income | 37.7 | 40.0 | 52.2 |
| Upper-middle-income | 61.4 | 34.8 | 79.0 |
| Lower-middle-income | 66.9 | 68.3 | 72.4 |
| Low-income | 72.1 | 75.0 | 70.0 |

*Notes. Income groups follow the four-level World Bank classification (high-, upper-middle-, lower-middle-, and low-income economies). For each group and content category (protocols, tools, videos), values are country-level medians of the proportion of sessions with a known device type that came from mobile devices (smartphones or tablets); only countries with at least one session in each category are included.*
