# Supplementary material for: Mobile-First Web Access and Captioned Video in Francophone Cardiology Education: Multicountry Ecological Learning Analytics Study
Source: JMIR Mhealth Uhealth. 2026 Sep 8;14:e90345. doi: 10.2196/90345 (PMC13552991; doi:10.2196/90345)
Supplement: Supplementary file 5 [file mhealth-v14-e90345-s005.docx]

**Table S2. Web referrer mix, LinkedIn activity, and website reach by income group (n=34 countries)**

| **Income group** | **Organic referrer share (%), median** | **Direct referrer share (%), median** | **Social referrer share (%), median** | **Other referrer share (%), median** | **LinkedIn followers (n), median** | **LinkedIn visitors (n/month), median** | **Unique website visitors (n), median** |
| --- | --- | --- | --- | --- | --- | --- | --- |
| High-income (HIC) | 33.0 | 39.6 | 14.3 | 0.62 | 17.0 | 14.5 | 278 |
| Middle-income (MIC) | 38.2 | 22.7 | 29.1 | 0.0 | 9.0 | 35.0 | 93 |
| Low-income (LIC) | 43.5 | 20.5 | 12.3 | 2.06 | 5.5 | NA | 43 |

*Notes. Referrer shares are percentages of ENC website sessions by referring source, summarized at the country level and expressed as medians within each income group. "Organic" refers to traffic from search engines; "Direct" to direct URL entry or bookmarks; "Social" to visits from social media platforms; "Other" includes email, referral links, and unclassified sources. LinkedIn followers and visitors are platform-reported counts; "NA" indicates that the value was not available from the LinkedIn analytics export for that group. Unique website visitors are country-level totals over the observation window.*
