# Supplementary material for: Mobile-First Web Access and Captioned Video in Francophone Cardiology Education: Multicountry Ecological Learning Analytics Study
Source: JMIR Mhealth Uhealth. 2026 Sep 8;14:e90345. doi: 10.2196/90345 (PMC13552991; doi:10.2196/90345)
Supplement: Supplementary file 6 [file mhealth-v14-e90345-s006.docx]

**Table S4. Web referrer mix by four-level World Bank income group**

| **Income group (four-level)** | **Organic referrer share (%), median** | **Direct referrer share (%), median** | **Social referrer share (%), median** | **Other referrer share (%), median** |
| --- | --- | --- | --- | --- |
| High-income | 33.0 | 39.6 | 14.3 | 0.62 |
| Upper-middle-income | 50.4 | 20.4 | 21.7 | 0.0 |
| Lower-middle-income | 37.2 | 23.1 | 29.4 | 0.0 |
| Low-income | 43.5 | 20.5 | 12.3 | 2.06 |

*Notes. Referrer categories are as defined in Supplementary Table S2. Values are country-level medians across countries within each four-level World Bank income group; not all countries had complete referrer data for every category.*
