# Supplementary material for: Mobile-First Web Access and Captioned Video in Francophone Cardiology Education: Multicountry Ecological Learning Analytics Study
Source: JMIR Mhealth Uhealth. 2026 Sep 8;14:e90345. doi: 10.2196/90345 (PMC13552991; doi:10.2196/90345)
Supplement: Supplementary file 7 [file mhealth-v14-e90345-s007.docx]

**STROBE Statement — Checklist of items that should be included in reports of cross-sectional studies**

*Mobile-First Web Access and Captioned Video in Francophone Cardiology Education: A Multi-Country Ecological Learning Analytics Study
(JMIR mHealth and uHealth, MS#90345 — revision May 2026)*

This checklist accompanies the revised manuscript "Mobile-First Web Access and Captioned Video in Francophone Cardiology Education: A Multi-Country Ecological Learning Analytics Study (n = 34 countries)." Page numbers refer to *02_Article ENCORE.docx* (clean version after Zotero bibliography pass). Section labels refer to Heading 1 / Heading 2 in that document. Multimedia Appendix 1 = this STROBE checklist. Supplementary Tables S1–S4 (four-level WB stratification, France-out sensitivity, referrer and content-category breakdowns) are listed in the main manuscript (Supplementary Tables section after the References); they are not separate upload files. The country-level scatterplot (Multimedia Appendix Figure S1) is uploaded as a separate Multimedia Appendix figure file. Items reported as "n/a — descriptive ecological design" reflect the absence of individual-level participants, exposures, follow-up, or matching, consistent with the country-level (group-level) design used.

| **Section / topic** | **Item #** | **Recommendation** | **Reported on (page / section)** |
| --- | --- | --- | --- |
| Title and abstract | 1(a) | Indicate the study's design with a commonly used term in the title or the abstract | Title (p.1) and Abstract / Methods (p.2-3) state: "cross-country observational ecological study (n = 34 countries)". |
|  | 1(b) | Provide in the abstract an informative and balanced summary of what was done and what was found | Structured Abstract (p.2-3): Background, Objectives, Methods, Results, Conclusions; balanced framing of effect sizes and absence of individual-level inference. |
| Introduction |  |  |  |
| Background/rationale | 2 | Explain the scientific background and rationale for the investigation being reported | Introduction (p.4): rationale for engagement-as-proxy in digital cardiology education across resource settings. |
| Objectives | 3 | State specific objectives, including any prespecified hypotheses | End of Introduction (p.4): three prespecified country-level objectives. No causal hypothesis claimed. |
| Methods |  |  |  |
| Study design | 4 | Present key elements of study design early in the paper | Methods / Study design (p.5): cross-country observational ecological design; unit of analysis = country. |
| Setting | 5 | Describe the setting, locations, and relevant dates, including periods of recruitment, exposure, follow-up, and data collection | Methods / Setting and data sources (p.5): École Numérique de Cardiologie web and YouTube analytics, two-year window 2023–2025; 34 countries. |
| Participants | 6(a) | Cross-sectional study—Give the eligibility criteria, and the sources and methods of selection of participants | Methods / Inclusion (p.5-6): all countries with non-zero web sessions and ≥ 1 ENC YouTube view in the period; 34 included. |
| Variables | 7 | Clearly define all outcomes, exposures, predictors, potential confounders, and effect modifiers. Give diagnostic criteria, if applicable | Methods / Variables (p.6): primary outcomes (mobile share, bounce rate, time on site, AVD, retention, intentional views); exposure (World Bank income group, three-level); descriptive secondary metrics (captions, demographics) explicitly framed as group-level summaries only. |
| Data sources/measurement | 8* | For each variable of interest, give sources of data and details of methods of assessment (measurement). Describe comparability of assessment methods if there is more than one group | Methods / Data sources (p.5–6): platform-native exports (GA4, YouTube Studio); definitions and computational rules tabulated. |
| Bias | 9 | Describe any efforts to address potential sources of bias | Methods / Limitations and bias (p.6–7, 18) + Discussion / Limitations (p.16–18): platform-attribution caveats, small-aggregate caveats for LIC YouTube demographics, ecological-fallacy explicit acknowledgement. |
| Study size | 10 | Explain how the study size was arrived at | Methods / Sample (p.5-6): convenience sample of all eligible countries with ENC analytics over the period; n = 34 fixed by data availability. |
| Quantitative variables | 11 | Explain how quantitative variables were handled in the analyses. If applicable, describe which groupings were chosen and why | Methods / Statistical analysis (p.6-7): medians + IQR for skewed metrics; income groups collapsed into three levels (HIC/MIC/LIC) for primary analyses; four-level analyses moved to Multimedia Appendix 1. |
| Statistical methods | 12(a) | Describe all statistical methods, including those used to control for confounding | Methods / Statistical analysis (p.7): nonparametric Kruskal–Wallis and Jonckheere–Terpstra trend tests; Spearman correlations at the country level only; quantile regression at τ=0.5 (Table 3); no inferential country-level claims on captions/demographics. |
|  | 12(b) | Describe any methods used to examine subgroups and interactions | Methods / Sensitivity analyses (p.7): four-level WB grouping in Multimedia Appendix 1; sensitivity analysis excluding France for HIC dominance. |
|  | 12(c) | Explain how missing data were addressed | Methods / Missing data (p.7-8): pairwise complete cases per analysis; n reported per row in Table 2 and Panel B. |
|  | 12(d) | Cross-sectional study—If applicable, describe analytical methods taking account of sampling strategy | n/a — full census of eligible ENC countries for the period (no sampling weights). |
|  | 12(e) | Describe any sensitivity analyses | Supplementary Tables S1–S4 (within the main manuscript): four-level WB stratification, France-out sensitivity. |
| Results |  |  |  |
| Participants | 13(a) | Report numbers of individuals at each stage of study | Results / Sample description (p.9) + Figure 1 (world map): 34 countries (13 HIC, 14 MIC, 7 LIC). |
|  | 13(b) | Give reasons for non-participation at each stage | n/a — country-level census of countries with ENC analytics. |
|  | 13(c) | Consider use of a flow diagram | Figure 1 (world map by WB group) serves as a sample-flow visualisation. |
| Descriptive data | 14(a) | Give characteristics of study participants and information on exposures and potential confounders | Results / Sample description (p.9) + Table 1 Panels A, A bis, B (p.10); demographic and caption metrics presented as group-level descriptive contrasts only. |
|  | 14(b) | Indicate number of participants with missing data for each variable of interest | Table 2 reports n per pair; Panel B reports n_countries_web; YouTube demographic n is small in LIC and explicitly flagged in the table footnote. |
| Outcome data | 15* | Cross-sectional study—Report numbers of outcome events or summary measures | Results / Primary engagement outcomes (p.9-10) + Tables 1B, 2 + Figure 3. |
| Main results | 16(a) | Give unadjusted estimates and, if applicable, confounder-adjusted estimates and their precision | Tables 1B, 2, 3; Figure 3 reports medians + IQR; quantile regression coefficients with 95% CI in Table 3. |
|  | 16(b) | Report category boundaries when continuous variables were categorized | Methods / Variables (p.5–6): three-level WB income grouping defined per current WB classification. |
|  | 16(c) | If relevant, translate estimates of relative risk into absolute risk for a meaningful time period | n/a — no relative-risk metric reported. |
| Other analyses | 17 | Report other analyses done—e.g. analyses of subgroups and interactions, and sensitivity analyses | Multimedia Appendix 1: four-level WB stratification, France-out sensitivity, Suppl. Figure S1 (country-level scatter). |
| Discussion |  |  |  |
| Key results | 18 | Summarise key results with reference to study objectives | Discussion / Principal findings (p.16): three group-level patterns aligned to the three prespecified objectives. |
| Limitations | 19 | Discuss limitations of the study, taking into account sources of potential bias or imprecision. Discuss both direction and magnitude of any potential bias | Discussion / Limitations (p.18): ecological fallacy, engagement ≠ learning outcomes, platform-attribution noise, small LIC YouTube aggregates, no individual-level inference. |
| Interpretation | 20 | Give a cautious overall interpretation of results considering objectives, limitations, multiplicity of analyses, results from similar studies, and other relevant evidence | Discussion / Interpretation (p.16-17): non-causal language; framed as descriptive ecological signal aligned with prior literature on mobile-first access in LMIC contexts. |
| Generalisability | 21 | Discuss the generalisability (external validity) of the study results | Discussion / Generalisability (p.17): single-publisher cohort (ENC); generalisability discussed across cardiology digital education ecosystems with similar mobile penetration profiles. |
| Other information |  |  |  |
| Funding | 22 | Give the source of funding and the role of the funders for the present study and, if applicable, for the original study on which the present article is based | Funding section (p.23): no external funding; ENC infrastructure self-financed; full disclosure included. |

*Notes. * Indicates information that should be given separately for cases and controls in case–control studies, or for exposed and unexposed groups in cohort and cross-sectional studies. The full STROBE Statement is available at https://www.strobe-statement.org. This checklist follows the cross-sectional version and was completed for the ENCORE country-level ecological design.*
